# Iron Status is Associated with Asthma and Lung Function in US Women

**DOI:** 10.1371/journal.pone.0117545

**Published:** 2015-02-17

**Authors:** Emily P. Brigham, Meredith C. McCormack, Clifford M. Takemoto, Elizabeth C. Matsui

**Affiliations:** 1 Johns Hopkins University School of Medicine, Division of Pulmonary and Critical Care Medicine, Baltimore, Maryland, United States of America; 2 Johns Hopkins University Bloomberg School of Public Health, Baltimore, Maryland, United States of America; 3 Johns Hopkins University School of Medicine, Division of Pediatric Hematology, Baltimore, Maryland, United States of America; 4 Johns Hopkins University School of Medicine, Division of Pediatric Allergy and Immunology, Baltimore, Maryland, United States of America; Lady Davis Institute for Medical Research/McGill University, CANADA

## Abstract

**Background:**

Asthma and iron deficiency are common conditions. Whether iron status affects the risk of asthma is unclear.

**Objective:**

To determine the relationship between iron status and asthma, lung function, and pulmonary inflammation.

**Methods:**

Relationships between measures of iron status (serum ferritin, serum soluble transferrin receptor (sTfR), and sTfR/log10ferritin (sTfR-F Index)) and asthma, lung function, and pulmonary inflammation were examined in women 20-49 years in the National Health and Nutrition Examination Survey. Logistic, linear, and quadratic regression models accounting for the survey design of NHANES were used to evaluate associations between iron status and asthma-related outcomes and were adjusted for race/ethnicity, age, smoking status, income, and BMI.

**Results:**

Approximately 16% reported a lifetime history of asthma, 9% reported current asthma, and 5% reported a recent asthma episode/attack (n = 2906). Increased ferritin (iron stores) was associated with decreased odds of lifetime asthma, current asthma, and asthma attacks/episodes in the range of ferritin linearly correlated with iron stores (20-300ng/ml). The highest quintile of ferritin (>76 ng/ml) was also associated with a decreased odds of asthma. Ferritin levels were not associated with FEV1. Increased values of the sTfR-F Index and sTfR, indicating *lower* body iron and *higher* tissue iron need, respectively, were associated with decreased FEV1, but neither was associated with asthma. None of the iron indices were associated with FeNO.

**Conclusion:**

In US women, higher *iron stores* were inversely associated with asthma and lower *body iron* and higher *tissue iron need* were associated with lower lung function. Together, these findings suggest that iron status may play a role in asthma and lung function in US women.

## Introduction

Asthma is one of the most common diseases in the United States. One in 11 children and 1 in 12 adults have asthma, and this disease resulted in 10.5 million missed school days and 14.2 million missed work days in 2008. The United States spends $56 billion annually on asthma medications [1]. Iron deficiency is also one of the most common nutritional deficiencies in the US [2,3] and is thought to be more prevalent in populations at greatest risk for asthma [3,4], suggesting that these two conditions may be linked.

While the role that iron plays in asthma is unknown, a physiologic link to asthma and inflammatory diseases like asthma has been suggested in animal models. A low iron diet resulted in pronounced asthma in a mouse model of allergic asthma, and this effect appeared to be mediated by increased mast cell reactivity in the setting of low iron [5]. Because these observations suggest that poor iron status could promote asthma, we hypothesized that lower iron would be associated with a greater risk of asthma, greater pulmonary inflammation, and decreased lung function. To test this hypothesis, we used iron indices and asthma data collected from women participating in the National Health and Nutrition Survey (NHANES) from 2007–2010.

## Methods

### Study Population

NHANES is a recurring survey of the non-institutionalized civilian US population (http://www.cdc.gov/nchs/nhanes/nhanes_questionnaires.htm). For this analysis, data from the 2007–2008 and 2009–2010 surveys were used because these years captured measures of iron status, lung function, and exhaled nitric oxide, as well as asthma outcomes. These data were only captured by NHANES in females 12–49 years of age. Because smoking status, a potentially important confounder, was best characterized in participants 20 years and older, our primary analysis was limited to adult females, ages 20–49 years. Therefore our primary sample is representative of the United States female, civilian, non-institutionalized population ages 20–49 years.

### Iron Indices

Serum ferritin, a measure of *iron stores*, was measured via the Roche/Hitachi 912 immunoturbidimetric assay in 2007–2008 and the Roche Elecsys 170 (E170) sandwich immunoassay in 2009–2010. In order to combine ferritin data that were measured using two different assays, the 2009–10 ferritin values were converted to values equivalent to the 2007–8 values using a publicly available equation derived from a Deming regression analysis [6]. Since ferritin has some inherent limitations as a measure of iron stores [7,8], relationships between ferritin and asthma-related outcomes were examined both across the full range of ferritin and within the range of ferritin that is most strongly and linearly correlated with iron stores (20–300ng/ml) [9–12].

Serum transferrin receptor (sTfR), which is a measure of *tissue iron need* [13], was also measured in NHANES. sTfR was measured via the Roche Hitachi 912 immunoturbidimetric assay in 2007–2008 and the Roche Hitachi Mod P immunoturbidimetric method in 2009–2010. If values of either ferritin or serum transferrin receptor were below the limit of detection, values were assigned as the lower limit of detection divided by the square root of two. A sTfR-F Index, which estimates *total body iron*, was calculated with the following equation, as previously reported [14]: sTfR/log_10_(ferritin). Increasing levels indicate increasing iron insufficiency.

### Asthma and Lung Function

To assess asthma prevalence, participants were asked whether a physician had ever diagnosed them with asthma, whether they still had asthma, and whether they had an asthma attack or episode within the prior year.

Spirometry was conducted in eligible participants, which were those without a prohibitive medical condition (current chest pain, physical problem with forced expiration, supplemental oxygen, recent eye, chest, or abdominal surgery, recent heart attack, stroke, tuberculosis exposure or coughing up blood, history of detached retina or collapsed lung). Quality attributes were assigned to each FEV_1_ and FVC, and those that were reported as “questionable” or “results not valid” were excluded from the analysis. No quality attributes specific to the FEF 25–75% were available, and all values were included. Percent predicted values were calculated using Hankinson et al. (1999) prediction equations [15].

Fractional exhaled nitric oxide (FeNO) was measured with the Aerocrine NIOX MINOⓇ(Aerocrine AB, Solna, Sweden) in eligible participants, which were those without a prohibitive medical condition (current chest pain, a physical problem with forceful expiration, using supplementary oxygen). The mean of two reproducible measurements was used for analysis. A reproducible measurement was defined as either below 30 ppb and within 2ppb of each other, or above 30 ppb and within 10% of each other. Two data points that were below the limit of detection were assigned a value of 3.5ppb, which is the lower limit of normal detection divided by the square root of 2. Two data points that were above the upper limit of detection (300ppb) were assigned a value of 301ppb.

### Statistical Analyses

All analyses used survey methods to account for the sampling scheme in NHANES and produce estimates representative of women ages 20–49 years in the US population. Ferritin and transferrin receptor concentrations, sTfR-F Index, as well as FeNO results, were log_10_-transformed. Linear and logistic regression analyses were performed to examine associations between measures of iron status and lung function, FeNO, and asthma. Potential confounders thought to be associated with iron status and asthma were included in multivariable models and included age, race/ethnicity, smoking status, income, and body mass index (BMI). Ferritin was examined: (1) across its full range, (2) in the range linearly correlated with iron stores (20–300 ng/ml inclusive), and (3) as quintiles. The quintile analyses were performed to explore the linearity of the relationships between ferritin and the outcomes of interest. Exploratory analyses suggested non-linear relationships between the full range of ferritin and some of the outcomes, therefore quadratic regression models were also applied. Sensitivity analyses were conducted by removing subjects with evidence of inflammation, which can elevate serum ferritin levels (defined as a total white blood cell count >10,000 cells/mm^3^ or C-reactive protein > 6.0 mg/dl) [16]. In addition, we investigated the combined effects of anemia and iron-deficiency on the outcomes of interest by categorizing anemia as a hemoglobin ≤12.0 g/dl (per World Health Organization Program Guidelines [17]) and iron deficiency as ferritin <20 ng/ml. All analyses were conducted with Stata SE (Version 11.2, StataCorp, College Station, TX). A two-tailed p-value <0.05 was considered statistically significant.

## Results

### Study Population Characteristics

The sample population was composed of females 20–49 years of age who participated in NHANES between 2007 and 2010. Survey-weighted characteristics therefore reflect the characteristics of non-institutionalized females 20–49 years in the US during this time period (Table 1, n = 2906). Approximately 16% of this US subpopulation ever had asthma, 9.1% had current asthma, and 5% had an asthma attack in the past year. Mean values for lung function indices and pulmonary inflammation, as captured by FeNO, were all within normal limits, as expected for a US population-based sample. The mean ferritin, an indicator of *iron stores*, was 36.4 ng/ml (SD: 0.7) and the mean sTfR, an indicator of *tissue iron need*, was 3.1 mg/L (SD: 0.03). Just under 25% had a ferritin less than 20 ng/mL, suggestive of deficient iron stores [18–20]. Cut points for quintiles of ferritin (ng/ml) as follows: Q1: 1.8–15.7; Q2: 16.0–28.7; Q3: 29.0–46.6; Q4: 47.0–76.0; and Q5: 76.8–1051.2. Within the fifth quintile, only 3% of values were above 300 ng/ml.

**Table 1:** Population characteristics (n = 2906).

**Socio-demographic Characteristics**	
Age (years)	35.0 (0.2)
Race/Ethnicity, % (SE)	
Non-Hispanic white	63.3 (3.0)
Non-Hispanic black	12.9 (1.3)
Mexican American	9.9 (1.4)
Other race or multiracial	7.3 (1.0)
Other Hispanic	6.5 (1.1)
Income to poverty ratio^§^	2.8 (0.07)
Smoking status, % (SE)	
Current	23.7 (1.4)
Former	14.3 (0.8)
Never	62.0 (1.9)
**Asthma**	
Lifetime, % (SE)	16.1 (0.9)
Current, % (SE)	9.1 (0.7)
Attack/Episode in Past Year, % (SE)	5.0 (0.5)
**Pulmonary Function/Inflammation**	
FEV_1_ ^†^	
Actual value (ml)	3027.2 (16.1)
% predicted	98.5 (0.4)
FVC ^$^	
Actual value (ml)	3739.1 (18.5)
% predicted	101.3 (0.3)
FEF25/75 ^‡^	
Actual value (ml/s)	3109.2 (26.0)
% predicted	94.1 (0.7)
FEV_1_/FVC ratio^ᶿ^	0.81 (0.002)
eNO (ppb)^ᶲ^	11.4 (0.3)
**Measures of Iron Status**	
Ferritin (ng/ml)	36.4 (0.7)
Soluble transferrin receptor (mg/L)^λ^	3.1 (0.03)
sTfR/log_10_(ferritin)^λ^	2.1 (0.02)
Iron deficient,* % (SE)	23.3 (0.8)

All data posted as mean (SE) unless otherwise indicated. Geometric means are presented for all variables log-transformed in presented analyses.

*ferritin < 20ng/ml

SE = linearized standard error

^§^n = 2678;

^†^n = 2415;

^$^n = 2378;

^‡^n = 2444;

^ᶿ^n = 2374;

^ᶲ^n = 2470;

^λ^n = 2901

### Relationships Between Iron Status and Asthma

Because ferritin is linearly correlated with iron stores in the range from 20–300 ng/ml, relationships between ferritin and asthma were examined in this range of ferritin. Models restricted to the range linearly related to iron stores (20–300 ng/ml) were created, and indicated that a ten-fold increase in ferritin within this range was associated with a 39% decrease in the odds of lifetime asthma (OR [95% CI]: 0.61 [0.39 to 0.95]), a 53% decrease in the odds of current asthma (OR [95% CI]: 0.47 [0.27 to 0.81]) and a 62% decrease in the odds of an asthma attack in the past year (OR [95% CI]: 0.38 [0.17 to 0.86]) adjusted for race/ethnicity, age, smoking, income, and BMI.

Analyses of relationships between ferritin quintiles created using the entire range of ferritin values and asthma demonstrated significantly decreased odds of all asthma outcomes for those in the highest ferritin quintile (>76ng/ml) compared to those in the lowest four quintiles (1.8–76 ng/ml) (Table 2). Results of analyses showing relationships between each ferritin quintile and asthma outcomes are available in S1 Table. Sensitivity analyses excluding the lowest quintile (ferritin <16.0 ng/ml, all iron deficient), and removing subjects with evidence of inflammation (defined as a total white blood cell count >10,000 cells/mm^3^ or C-reactive protein > 6.0 mg/dl) did not appreciably affect results (data not shown).

**Table 2:** Relationships between iron status and asthma outcomes.

	Lifetime Asthma		Current Asthma		Asthma Attack/Episode in Past Year	
	Unadjusted	Adjusted*	Unadjusted	Adjusted*	Unadjusted	Adjusted*
	OR (95% CI)		OR (95% CI)		OR (95% CI)	
**Ferritin Iron Indices (higher levels indicative of *more* iron)**						
Log_10_(ferritin)^‡^	0.89 (0.69 to 1.16)	0.81 (0.64 to 1.04)	0.90 (0.66 to 1.23)	0.78 (0.57 to 1.04)	1.03 (0.74 to 1.45)	0.84 (0.65 to 1.09)
Log_10_(ferritin)^§^_(20–300 ng/ml)_	**0.61 (0.39 to 0.94)**	**0.61 (0.39 to 0.95)**	**0.59 (0.36 to 0.97)**	**0.47 (0.27 to 0.81)**	0.65 (0.30 to 1.42)	**0.38 (0.17 to 0.86)**
Ferritin, quintile 5 vs. quintiles 1–4^‡^	0.77 (0.58 to 1.02)	**0.69 (0.52 to 0.91)**	0.84 (0.61 to 1.15)	**0.64 (0.45 to 0.92)**	0.85 (0.55 to 1.32)	**0.56 (0.38 to 0.84)**
**Serum Transferrin Receptor Iron Indices (higher levels indicative of *less* iron)**						
Log_10_(sTFR)^†^	1.36 (0.83 to 2.21)	1.30 (0.76 to 2.21)	1.61 (0.73 to 3.58)	1.15 (0.46 to 2.85)	2.13 (0.73 to 6.18)	1.67 (0.47 to 5.98)
Log_10_(sTfR/log_10_ ferritin)^†^	1.14 (0.82 to 1.58)	1.20 (0.86 to 1.66)	1.22 (0.76 to 1.95)	1.18 (0.72 to 1.92)	1.26 (0.71 to 2.25)	1.27 (0.69 to 2.37)

*Adjusted for race/ethnicity, age, smoking, income, and BMI

**Bolded** results are statistically significant, with p<0.05

^‡^n = 2906 for unadjusted, n = 2663 for adjusted; ferritin Q1–4: 1.8–76.0ng/ml, Q5: >76.0ng/ml

^§^Ferritin restricted to values from 20 to 300 ng/ml, inclusive; n = 2134 for unadjusted, n = 1963 for adjusted

^†^n = 2901 for unadjusted, n = 2658 for adjusted

**Fig 1:**
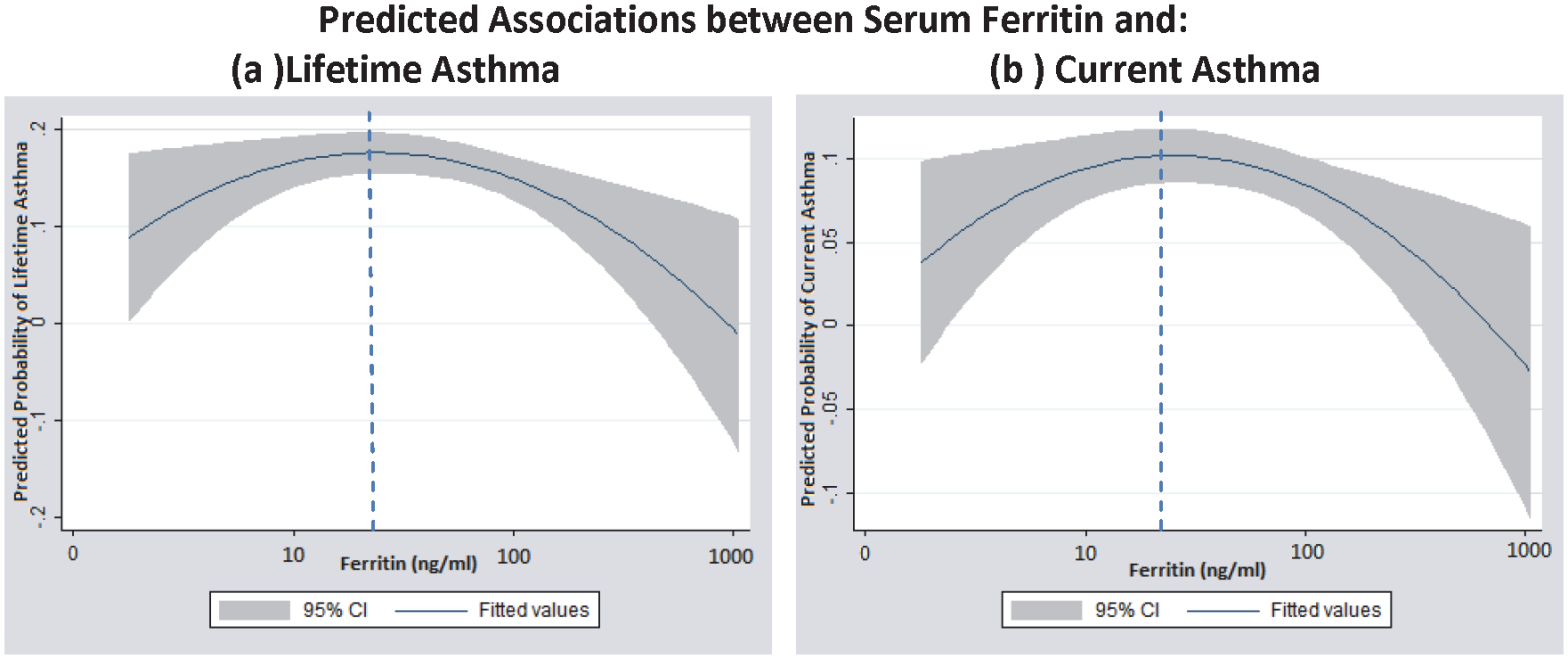
Predicted relationships between the full range of ferritin concentrations and asthma- related outcomes, generated from regression models: (a) lifetime asthma and (b) current asthma. 95% confidence intervals are depicted by the shaded areas. Vertical line represents approximately 20ng/ml ferritin.

Significant relationships between the entire range of ferritin and asthma were not evident in a linear prediction model (Table 2). However, there was evidence to support non-linear relationships between ferritin and asthma outcomes (Fig. 1). Quadratic models demonstrated significant, non-linear relationships between ferritin and lifetime and current asthma (quadratic term p-values <0.05) and a trend towards a non-linear relationship with asthma attack/episode in the past year (quadratic term p-value = 0.10) (S2 Table). Specifically, at levels of ferritin that reflect deficient iron stores (<20–30 ng/ml), the odds of lifetime and current asthma increased with increasing ferritin levels (Fig. 1). However, as ferritin levels increased from levels reflective of insufficient iron stores (<20–30 ng/ml) to levels reflective of sufficient iron stores, the odds of these asthma outcomes decreased.

Neither sTfR (*tissue iron need*) nor the sTfR-F Index (*total body iron*) were associated with the asthma outcomes. Results are shown in Table 2.

### Relationships Between Iron Status and Lung Function and Pulmonary Inflammation

Ten-fold increases in sTfR (*tissue iron need*) or the sTfR-F Index (*total body iron*) were associated with a significant decrease in FEV_1_ percent predicted (β coefficient [95% CI]: -4.5 [-8.2 to-0.8]) and-2.1 [-4.2 to-0.1], respectively) (Table 3). Increases in both sTfR (*tissue iron need*) and the sTfR-F Index (*total body iron*) were also associated with significant decreases in FEF 25–75 percent predicted (β coefficient [95% CI]: -8.5 [-14.5 to-2.4] and-5.1 [-8.7 to-1.4], respectively). These relationships were consistently present in models using actual rather than percent predicted spirometric values (S3 Table). Neither sTfR (*tissue iron need*) nor the sTfR-F Index (*total body iron*) was associated with FeNO levels.

**Table 3:** Relationships between iron status and lung function and FeNO.

	FEV_1_/FVC ratio	FEV_1_% predicted	FVC % predicted	FEF 25–75% predicted	Log_10_(FeNO)
	β (95% CI)	β (95% CI)	β (95% CI)	β (95% CI)	β (95% CI)
**Ferritin Iron Indices (higher levels indicative of *more* iron)**					
Log_10_(ferritin)^‡^	**0.009 (0.003 to 0.01)**	0.2 (-1.0 to 1.4)	-0.8 (-2.0 to 0.4)	2.5 (-0.03 to 5.1)	-0.01 (-0.05 to 0.03)
Log_10_ (ferritin)^§^_(20–300 ng/ml)_	**0.01 (0.004 to 0.03)**	-1.9 (-4.8 to 0.9)	**-3.4 (-6.4 to-0.3)**	3.5 (-1.3 to 8.3)	-0.05 (-0.1 to 0.01)
Ferritin, quintile 5 vs. quintiles 1–4^‡^	**0.01 (0.002 to 0.02)**	-0.1 (-1.5 to 1.3)	-1.1 (-2.7 to 0.5)	2.4 (-0.7 to 5.6)	-0.03 (-0.06 to 0.004)
**Serum Transferrin Receptor Iron Indices (higher levels indicative of *less* iron)**					
Log_10_ (sTFR)^†^	-0.02 (-0.04 to 0.0008)	**-4.5 (-8.2 to-0.8)**	-2.6 (-7.1 to 1.8)	**-8.5 (-14.5 to-2.4)**	0.03 (-0.05 to 0.11)
Log_10_ (sTfR/log_10_ ferritin)^†^	**-0.01 (-0.02 to-0.002)**	**-2.1 (-4.2 to-0.1)**	-0.8 (-3.2 to 1.7)	**-5.1 (-8.7 to-1.4)**	0.02 (-0.03 to 0.07)

Adjusted for race/ethnicity, age, smoking, income, and BMI

**Bolded** results are statistically significant, with p<0.05

^‡^FEV_1_/FVC % (n = 2198); FEV_1_% predicted (n = 2236); FVC (n = 2201); FEF 25–75% predicted (n = 2261); Log_10_(FeNO) (n = 2279); ferritin Q1–4: 1.8–76.0ng/ml, Q5: >76.0ng/ml

^§^Ferritin restricted to values from 20 to 300 ng/ml, inclusive; FEV_1_/FVC % (n = 1630); FEV_1_% predicted (n = 1657); FVC (n = 1633); FEF 25–75% predicted (n = 1676); Log_10_(FeNO) (n = 1686)

^†^FEV_1_/FVC % (n = 2195); FEV_1_% predicted (n = 2233); FVC (n = 2198); FEF 25–75% predicted (n = 2258); Log_10_(FeNO) (n = 2276)

Overall, higher ferritin levels were not associated with better lung function or lower FeNO levels (Table 3; S4 and S5 Tables). Although ferritin across the full range was associated with a higher FEV_1_/FVC ratio, this finding was explained by the association between higher ferritin levels and a lower FVC (Table 3). As expected, similar relationships were present in models using actual rather than percent predicted spirometric values (S3 Table).

### Iron Deficiency, Anemia, and Asthma-related Outcomes

We examined the effects of iron deficiency with and without anemia on asthma, lung function, and pulmonary inflammation. There were no associations between iron deficiency with or without anemia and asthma outcomes, lung function, or pulmonary inflammation when comparing iron-sufficient, non-anemic individuals to any of the other categories (S6 and S7 Tables).

### Secondary Analyses: Age 12–19

As a secondary analysis of the data available in NHANES, we examined the above relationships in adolescent females age 12–19. We found a significant, positive relationships between sTfR (*tissue iron need*) and asthma attack (OR [95% CI]: 6.13 [1.16 to 32.43]) and sTfR-F Index (*total body iron*) and asthma attack (OR [95% CI]: 2.97 [1.01 to 8.71]), consistent with the results in adult females (S8 Table). We did not observe any relationships between ferritin and asthma outcomes among adolescent females. However, it is important to note that the distribution of ferritin was lower in this younger age group than in the adult female population, as only 6.4% of adolescent females had a ferritin greater than 76 ng/ml, the level found to be protective in the adult population. Furthermore, sample size in adolescents (n = 1046) was roughly one third that of adults (n = 2906), so that statistical power was lower in the adolescent analyses than the adult women analyses. We did not find meaningful relationships between any of the iron indices and lung function in these analyses restricted to adolescent females (data not shown).

## Discussion

We found that higher iron stores, as represented by higher serum ferritin, were associated with a lower prevalence of asthma in US women 20–49 years of age. Specifically, ferritin >76 ng/ml was associated with decreased odds of lifetime asthma, current asthma and asthma attack/episode in the past year. Notably, increases in tissue iron need and decreases in body iron, (represented by lower sTfR and lower sTfR-F Index, respectively) were linked to decreases in FEV_1_, suggesting that tissue and body iron may influence lung function. Together, these findings suggest that iron status could have significant effects on pulmonary physiology and the risk of asthma. Given that suboptimal iron status and asthma are both common in the United States, a better understanding of the relationship between iron and asthma could provide an opportunity to intervene on asthma through iron supplementation.

While poor neurodevelopmental outcomes and cancer prognosis have been linked to iron deficiency, [21–23], links between iron status and pulmonary disease remain relatively unexplored. However, some recent studies suggest that iron status may affect the lungs. In one study, iron supplementation in iron deficient women (average serum ferritin of 9.3 ng/ml at baseline, increased to an average of 42.9 ng/ml after supplementation) led to resolution of chronic cough and bronchial hyperreactivity [24]. Notably, forty-one percent of these women had normal hemoglobin levels at baseline prior to supplementation, implicating iron repletion as the primary factor in their symptom improvement. In a study of children in India, anemia, which was predominantly due to iron deficiency, was associated with asthma [25]. Lastly, a birth cohort study found an inverse association between umbilical cord iron levels and later onset wheeze and eczema [26]. Iron deficiency has also been associated with poorer functional status in patients with idiopathic pulmonary hypertension (IPH) [27]. Because of this link between iron status and functional status in IPH and the observation that iron can decrease pulmonary arterial vasoconstriction [28,29], a clinical trial is planned to determine the effects of iron supplementation on IPH [30]. Together these studies lend credence to the general notion that iron status may have direct effects on the lungs, and are supportive of our findings demonstrating an association between iron status and asthma and lung function.

Our study’s findings are further supported by *in vitro* and mouse models, which point to the potential biological basis of the association between iron status and asthma-related outcomes. For example, mouse model studies have demonstrated that iron administration reduces airway eosinophilia and hyperreactivity [5,31]. One of these studies also demonstrated less production of pro-inflammatory cytokines in male mice fed an iron sufficient diet compared to male mice fed an iron deficient diet, but who had not progressed to anemia [5]. Iron stores were five to six times and higher in the liver and spleen of mice fed an iron sufficient diet as compared to an iron deficient diet, indicating that the differences seen were associated with actual induced differences in iron stores. The highest increase in cytokine level was noted in IL-17, a known mediator for asthma in humans [32]. Furthermore, mast cells incubated in low iron media had an increase in IgE-mediated degranulation [5]. As mast cells and eosinophils play important roles in the acute and chronic inflammation of asthma, the studies above support the biologic plausibility of a causal relationship between iron status, asthma, and lung function [33–35].

In our study, there was a clear and consistent association between the highest ferritin quintile (>76ng/ml) and decreased odds of all three asthma outcomes, suggesting that supplementation with iron aimed at increasing ferritin to a level above this threshold could be an experimental target for asthma risk reduction. Interestingly, in other disease states affected by iron stores, a similar threshold effect of ferritin has been observed. In restless leg syndrome and alopecia, for example, iron supplementation to achieve ferritin levels of at least 50 ng/ml [36] and 70 ng/ml, respectively, have been proposed [37,38].

Ferritin levels, however, were not associated with better lung function, and instead were associated with a lower FVC. As lower FVC is a feature of restrictive lung disease, this finding merits further investigation in future studies. The other two iron indices, sTfR (*tissue iron need*) and the sTfR-F Index (*total body iron*), were not associated with asthma outcomes, but were associated with FEV_1_ and FEF25–75. Specifically, higher *tissue iron need* and lower *total body iron* were associated with a lower FEV_1_ and a lower FEF 25–75, lung function findings that would be consistent with asthma, suggesting that iron status may influence lung function. Together, the associations between the iron indices and asthma and lung function support the hypothesis that iron may play a role in the initiation and/or perpetuation of asthma.

The nonlinear association between the full range of ferritin and asthma in our study is curious, but perhaps not without reason. For example, it is possible that at the low end of the ferritin/iron stores spectrum, those with frank iron deficiency are unable to mount a robust inflammatory response and therefore may be protected against asthma. As iron stores are repleted, immune cell function improves, the inflammatory response is restored, and this phenomenon could result in an increase in risk of asthma. This phenomenon of limited inflammatory response in iron deficiency, and restored inflammatory response with iron repletion is well described in the infectious disease literature [39–42], so it is plausible that this same phenomenon could mediate the relationships between iron and asthma that were observed among those whose ferritin levels were below 20–30ng/ml.

Ferritin is known to be an imperfect indicator of iron status, and without more invasive testing it is difficult to determine true body iron stores. However, this limitation was addressed by examining relationships with ferritin in the range of ferritin values that are strongly and linearly correlated with iron stores [9–12], as well as the full range of ferritin. We also examined sTfR, which reflects *tissue iron need*, and the sTfR-F Index, which is a robust measure of *total body iron* [43], and associations between these additional measures of iron status and asthma were consistent with those observed for ferritin. Since ferritin was most consistently and strongly associated with the asthma outcomes, it is likely that iron stores, rather than tissue iron or body iron, plays a more important role in determining asthma risk. It is also possible that the lack of significant relationships between sTfR and the asthma outcomes could be due to the fact that sTfR is most strongly related to iron status in the setting of low ferritin levels [13,43], so it may not accurately capture iron status for most of the study population, resulting in weak and non-significant relationships with asthma.

While our findings are novel and suggest that iron status deserves further study as a potential cause of asthma, there are several additional limitations that should be considered. As our sample includes women only, it is unknown whether these findings are applicable to men. It is also possible that our findings with respect to ferritin were confounded by inflammation since ferritin is an acute-phase reactant and is known to be elevated in inflammatory states. However, higher ferritin was associated with a lower risk of asthma, rather than a higher risk of asthma as would be expected if ferritin were simply serving as a marker of an inflammatory disease. Furthermore, sensitivity analyses removing subjects with evidence of systemic inflammation did not alter our results. Iron status may act as an indicator of nutritional status or aspects of diet not investigated or controlled for in these analyses, though the supplementation studies discussed above are suggestive of an independent effect. Lastly, in a cross-sectional study, reverse causality cannot be excluded, so it is possible that having asthma leads to lower iron stores, which should be evaluated in prospective studies.

In summary, we found that higher iron stores were inversely associated with asthma and higher *tissue iron need* and lower *total body iron* were associated with lower lung function in US women. These findings, which are biologically plausible, merit further investigation in longitudinal studies and in other study populations, which will inform the need for randomized controlled trials of iron supplementation for asthma prevention. Investigation of the role of iron status in asthma morbidity is also merited, given the findings in this study and the effect of iron on mast cells and eosinophils, which are known to play key roles in the asthmatic response. Should iron insufficiency prove to increase the risk of asthma, it would afford an opportunity to reduce the risk of asthma using an inexpensive intervention in a substantial number of individuals.

## Supporting Information

S1 Table Relationships between ferritin quintiles and asthma outcomes. *Adjusted for race/ethnicity, age, smoking, income, and BMI. n = 2906 for unadjusted, n = 2663 for adjusted.
(DOCX)

S2 Table Logistic and quadratic models of relationships between the full range of ferritin and asthma outcomes. All models adjusted for race/ethnicity, age, smoking, income, and BMI. **Bolded** results are statistically significant, with p<0.05. *n = 2663.
(DOCX)

S3 Table Relationships between iron status and actual PFT values. Adjusted for race/ethnicity, age, smoking, income, height, and BMI. **Bolded** results are statistically significant, with p<0.05. ^‡^ FEV_1_ (n = 2236); FVC (n = 2201); FEF 25–75 (n = 2261). ^§^Ferritin restricted to values from 20 to 300 ng/ml, inclusive; FEV_1_ (n = 1657); FVC (n = 1633); FEF25–75 (n = 1676). ^†^ FEV_1_ (n = 2233); FVC (n = 2198); FEF25–75 (n = 2258). ^§^Q1–4: 1.8–76.0ng/ml, Q5: >76.0ng/ml.
(DOCX)

S4 Table Linear and quadratic models of relationships between the full range of ferritin and lung function and FeNO. All models adjusted for race/ethnicity, age, income, BMI, and smoking. **Bolded** results are statistically significant, with p<0.05. *n = 2198; ^n = 2236; ^ᶲ^n = 2201; ^†^n = 2279.
(DOCX)

S5 Table Relationships between iron status and lung function and FeNO. Adjusted for race/ethnicity, age, smoking, income, and BMI. **Bolded** results are statistically significant, with p<0.05. FEV_1_/FVC (n = 2198); FEV_1_ (n = 2236); FVC (n = 2201); FEF 25–75 (n = 2261); Log_10_(FeNO) (n = 2279).
(DOCX)

S6 Table Relationships between iron deficiency, anemia, and asthma outcomes. *Adjusted for race/ethnicity, age, smoking, income, and BMI. Anemia was defined as a hemoglobin < 12.0 mg/dL and iron deficiency was defined as ferritin <20 ng/mL. n = 2900 for unadjusted, n = 2658 for adjusted.
(DOCX)

S7 Table Relationships between iron deficiency, anemia, lung function and inflammation. Adjusted for race/ethnicity, age, smoking, income, and BMI. Anemia was defined as a hemoglobin <12.0 mg/dL and iron deficiency was defined as ferritin <20 ng/mL. ^§^n = 2193; ^†^n = 2274.
(DOCX)

S8 Table Relationships between iron status and asthma outcomes. *Adjusted for race/ethnicity, age, smoking, income, and BMI. **Bolded** results are statistically significant, with p<0.05. ^‡^n = 1046 for unadjusted, n = 930 for adjusted; Q1–4: 1.8–48.0ng/ml, Q5: >48.0ng/ml. ^§^ferritin restricted to values from 20 to 300 ng/ml, inclusive; n = 693 for unadjusted, n = 621 for adjusted. ^†^n = 1043 for unadjusted, n = 927 for adjusted.
(DOCX)
