# Supplementary material for: Iron Status is Associated with Asthma and Lung Function in US Women
Source: PLoS One. 2015 Feb 17;10(2):e0117545. doi: 10.1371/journal.pone.0117545 (PMC4331366; doi:10.1371/journal.pone.0117545)
Supplement: S1 Table — *Adjusted for race/ethnicity, age, smoking, income, and BMI. n = 2906 for unadjusted, n = 2663 for adjusted. (DOCX) [file pone.0117545.s001.docx]

|  | **Lifetime Asthma** | | **Current Asthma** | | **Asthma Attack/Episode in Past Year** | |
| --- | --- | --- | --- | --- | --- | --- |
|  | Unadjusted | Adjusted* | Unadjusted | Adjusted* | Unadjusted | Adjusted* |
| **Ferritin** | OR (95% CI) | | OR (95% CI) | | OR (95% CI) | |
| Quintile 1 (1.8-15.7 ng/ml) | -ref- | -ref- | -ref- | -ref- | -ref- | -ref- |
| Quintile 2 (16-28.7 ng/ml) | 1.11 (0.78 to 1.57) | 1.08 (0.74 to 1.56) | 1.14 (0.68 to 1.88) | 1.25 (0.70 to 2.21) | 1.46 (0.78 to 2.75) | 1.78 (0.88 to 3.59) |
| Quintile 3 (29-46.6 ng/ml) | 0.95 (0.65 to 1.38) | 0.90 (0.62 to 1.31) | 0.97 (0.58 to 1.61) | 1.01 (0.59 to 1.72) | 1.22 (0.67 to 2.25) | 1.43 (0.75 to 2.73) |
| Quintile 4 (47-76 ng/ml) | 1.17 (0.83 to 1.66) | 1.12 (0.78 to 1.62) | 1.16 (0.74 to 1.81) | 1.20 (0.75 to 1.93) | 1.40 (0.82 to 2.36) | 1.52 (0.87 to 2.66) |
| Quintile 5 (76.8-1051.2 ng/ml) | 0.81 (0.54 to 1.21) | 0.71 (0.48 to 1.04) | 0.89 (0.53 to 1.50) | 0.72 (0.42 to 1.23) | 1.09 (0.63 to 1.91) | 0.81 (0.51 to 1.30) |

**Table S1.** Relationships between ferritin quintiles and asthma outcomes.

*Adjusted for race/ethnicity, age, smoking, income, and BMI

n=2906 for unadjusted, n=2663 for adjusted
