# Supplementary material for: Iron Status is Associated with Asthma and Lung Function in US Women
Source: PLoS One. 2015 Feb 17;10(2):e0117545. doi: 10.1371/journal.pone.0117545 (PMC4331366; doi:10.1371/journal.pone.0117545)
Supplement: S2 Table — All models adjusted for race/ethnicity, age, smoking, income, and BMI. Bolded results are statistically significant, with p<0.05. *n = 2663. (DOCX) [file pone.0117545.s002.docx]

**Table S2.** Logistic and quadratic models of relationships between the full range of ferritin and asthma outcomes.

|  | **Lifetime Asthma*** | | **Current Asthma*** | | **Asthma Attack/Episode in Past Yr*** | |
| --- | --- | --- | --- | --- | --- | --- |
|  | Linear Model | Quadratic Model | Linear Model | Quadratic Model | Linear Model | Quadratic Model |
|  | OR (95% CI) | | OR (95% CI) | | OR (95% CI) | |
| Log_10_(ferritin) | 0.81 (0.64 to 1.04) | **3.41 (1.09 to 10.68)** | 0.77 (0.57 to 1.04) | **8.65 (1.10 to 68.10)** | 0.84 (0.65 to 1.09) | 12.32 (0.46 to 327.19) |
| [Log_10_(ferritin)]^2^ | -- | **0.61 (0.42 to 0.90)** | -- | **0.44 (0.22 to 0.86)** | -- | 0.41 (0.14 to 1.22) |

All models adjusted for race/ethnicity, age, smoking, income, and BMI

**Bolded** results are statistically significant, with p<0.05

*n=2663
