# Supplementary material for: Iron Status is Associated with Asthma and Lung Function in US Women
Source: PLoS One. 2015 Feb 17;10(2):e0117545. doi: 10.1371/journal.pone.0117545 (PMC4331366; doi:10.1371/journal.pone.0117545)
Supplement: S3 Table — Adjusted for race/ethnicity, age, smoking, income, height, and BMI. Bolded results are statistically significant, with p<0.05. ‡ FEV1 (n = 2236); FVC (n = 2201); FEF 25–75 (n = 2261). §Ferritin restricted to values from 20 to 300 ng/ml, inclusive; FEV1 (n = 1657); FVC (n = 1633); FEF25–75 (n = 1676). † FEV1 (n = 2233); FVC (n = 2198); FEF25–75 (n = 2258). §Q1–4: 1.8–76.0ng/ml, Q5: >76.0ng/ml. (DOCX) [file pone.0117545.s003.docx]

**Table S3.** Relationships between iron status and actual PFT values.

|  | **FEV_1_ (ml)** | **FVC (ml)** | | **FEF 25-75 (ml/s)** |
| --- | --- | --- | --- | --- |
|  | β (95% CI) | β (95% CI) | | β (95% CI) |
| **Ferritin Iron Indices (higher levels indicative of *more* iron)** | | | | |
| Log_10_(ferritin)^‡^ | 8.2 (-31.0 to 47.4) | | -29.2 (-76.4 to 18.0) | **90.3 (8.4 to 172.2)** |
| Log_10_(ferritin)^§^ _(20-300 ng/ml)_ | -55.9 (-145.0 to 33.2) | | **-132.7 (-249.9 to -15.5)** | 128.6 (-26.8 to 283.9) |
| Ferritin, quintile 5 vs. quintiles 1-4^‡^ | 0.3 (-43.1 to 43.6) | | -44.3 (-106.7 to 18.1) | 91.9 (-7.8 to 191.5) |
| **Serum Transferrin Receptor Iron Indices (higher levels indicative of *less* iron)** | | | | |
| Log_10_ (sTFR)^†^ | **-136.2 (-251.8 to -20.6)** | | -104.7 (-274.2 to 64.8) | **-274.5 (-465.7 to -83.4)** |
| Log_10_ (sTfR/log_10_ ferritin)^†^ | **-66.4 (-132.0 to -0.9)** | | -31.0 (-123.6 to 61.7) | **-169.4 (-284.3 to -54.6)** |

Adjusted for race/ethnicity, age, smoking, income, height, and BMI

**Bolded** results are statistically significant, with p<0.05

^‡^ FEV_1_ (n=2236); FVC (n= 2201); FEF 25-75 (n=2261)

^§^Ferritin restricted to values from 20 to 300 ng/ml, inclusive; FEV_1_ (n=1657); FVC (n=1633); FEF25-75 (n=1676)

^†^ FEV_1_ (n=2233); FVC (n=2198); FEF25-75 (n=2258)

^§^Q1-4: 1.8-76.0ng/ml, Q5: >76.0ng/ml
