# Supplementary material for: Iron Status is Associated with Asthma and Lung Function in US Women
Source: PLoS One. 2015 Feb 17;10(2):e0117545. doi: 10.1371/journal.pone.0117545 (PMC4331366; doi:10.1371/journal.pone.0117545)
Supplement: S4 Table — All models adjusted for race/ethnicity, age, income, BMI, and smoking. Bolded results are statistically significant, with p<0.05. *n = 2198; ^n = 2236; ᶲn = 2201; †n = 2279. (DOCX) [file pone.0117545.s004.docx]

|  | **FEV_1_/FVC ratio*** | | **FEV_1_ % predicted**^^^ | | **FVC % predicted**^ᶲ^ | | **Log_10_(FeNO)**^†^ | |
| --- | --- | --- | --- | --- | --- | --- | --- | --- |
|  | Linear Model | Quadratic Model | Linear Model | Quadratic Model | Linear Model | Quadratic Model | Linear Model | Quadratic Model |
|  | β (95% CI) | | β (95% CI) | | β (95% CI) | | β (95% CI) | |
| Log_10_ (ferritin) | **0.009 (0.003 to 0.01)** | -0.007 (-0.05 to 0.04) | 0.18 (-0.99 to 1.35) | 8.53 (-0.25 to 17.31) | -0.77 (-1.98 to 0.44) | **9.08 (0.80 to 17.37)** | -0.01 (-0.05 to 0.03) | 0.11 (-0.07 to 0.28) |
| [Log_10_ (ferritin)]^2^ | -- | 0.005 (-0.009 to 0.02) | -- | -2.81 (-5.78 to 0.15) | -- | **-3.32 (-6.09 to -0.55)** | -- | -0.04 (-0.10 to 0.02) |

**Table S4.** Linear and quadratic models of relationships between the full range of ferritin and lung function and FeNO.

All models adjusted for race/ethnicity, age, income, BMI, and smoking

**Bolded** results are statistically significant, with p<0.05

*n=2198; ^n=2236; ^ᶲ^n=2201; ^†^n=2279
