# Supplementary material for: Iron Status is Associated with Asthma and Lung Function in US Women
Source: PLoS One. 2015 Feb 17;10(2):e0117545. doi: 10.1371/journal.pone.0117545 (PMC4331366; doi:10.1371/journal.pone.0117545)
Supplement: S5 Table — Adjusted for race/ethnicity, age, smoking, income, and BMI. Bolded results are statistically significant, with p<0.05. FEV1/FVC (n = 2198); FEV1 (n = 2236); FVC (n = 2201); FEF 25–75 (n = 2261); Log10(FeNO) (n = 2279). (DOCX) [file pone.0117545.s005.docx]

**Table S5.** Relationships between iron status and lung function and FeNO.

|  | **FEV_1_/FVC ratio** | **FEV_1_ % predicted** | **FVC % predicted** | **FEF 25-75 % predicted** | **Log_10_(FeNO)** |
| --- | --- | --- | --- | --- | --- |
| **Ferritin** | β (95% CI) | β (95% CI) | β (95% CI) | β (95% CI) | β (95% CI) |
| Quintile 1 (1.8-15.7 ng/ml) | -ref- | -ref- | -ref- | -ref- | -ref- |
| Quintile 2 (16-28.7 ng/ml) | 0.003 (-0.004 to 0.01) | 0.48 (-1.46 to 2.41) | -0.05 (-2.05 to 1.96) | 0.15 (-2.93 to 3.23) | 0.03 (-0.02 to 0.07) |
| Quintile 3 (29-46.6 ng/ml) | -0.00002 (-0.01 to 0.01) | 1.31 (-0.81 to 3.42) | 1.42 (-0.86 to 3.70) | 0.93 (-3.03 to 4.90) | 0.03 (-0.02 to 0.07) |
| Quintile 4 (47-76 ng/ml) | 0.005 (-0.003 to 0.01) | 0.15 (-1.89 to 2.19) | -0.49 (-2.58 to 1.60) | 1.46 (-1.92 to 4.83) | 0.007 (-0.04 to 0.05) |
| Quintile 5 (76.8-1051.2 ng/ml) | **0.01 (0.003 to 0.02)** | 0.39 (-1.23 to 2.02) | -0.87 (-2.66 to 0.92) | 3.09 (-0.59 to 6.78) | -0.01 (-0.06 to 0.03) |

Adjusted for race/ethnicity, age, smoking, income, and BMI

**Bolded** results are statistically significant, with p<0.05

FEV_1_/FVC (n=2198); FEV_1_ (n=2236); FVC (n=2201); FEF 25-75 (n=2261); Log_10_(FeNO) (n=2279)
