# Supplementary material for: Iron Status is Associated with Asthma and Lung Function in US Women
Source: PLoS One. 2015 Feb 17;10(2):e0117545. doi: 10.1371/journal.pone.0117545 (PMC4331366; doi:10.1371/journal.pone.0117545)
Supplement: S6 Table — *Adjusted for race/ethnicity, age, smoking, income, and BMI. Anemia was defined as a hemoglobin < 12.0 mg/dL and iron deficiency was defined as ferritin <20 ng/mL. n = 2900 for unadjusted, n = 2658 for adjusted. (DOCX) [file pone.0117545.s006.docx]

**Table S6.** Relationships between iron deficiency, anemia, and asthma outcomes.

|  |  | **Lifetime Asthma** | | **Current Asthma** | | **Asthma Attack/Episode in Past Year** | |
| --- | --- | --- | --- | --- | --- | --- | --- |
|  |  | Unadjusted | Adjusted* | Unadjusted | Adjusted* | Unadjusted | Adjusted* |
|  | **Iron status** | OR (95% CI) | | OR (95% CI) | | OR (95% CI) | |
| **No anemia** | Sufficient | -ref- | -ref- | -ref- | -ref- | -ref- | -ref- |
|  | Deficient | 0.91 (0.65 to 1.26) | 0.97 (0.69 to 1.37) | 0.94 (0.60 to 1.46) | 0.96 (0.61 to 1.53) | 0.85 (0.48 to 1.48) | 0.84 (0.47 to 1.52) |
| **Anemia** | Sufficient | 0.91 (0.51 to 1.63) | 0.94 (0.53 to 1.67) | 0.95 (0.38 to 2.34) | 0.79 (0.32 to 1.93) | 0.95 (0.34 to 2.68) | 0.95 (0.30 to 3.05) |
|  | Deficient | 0.84 (0.55 to 1.28) | 0.95 (0.64 to 1.40) | 0.82 (0.53 to 1.28) | 0.90 (0.56 to 1.45) | 0.85 (0.46 to 1.55) | 0.99 (0.53 to 1.85) |

*Adjusted for race/ethnicity, age, smoking, income, and BMI

Anemia was defined as a hemoglobin < 12.0 mg/dL and iron deficiency was defined as ferritin <20 ng/mL

n=2900 for unadjusted, n=2658 for adjusted
