# Supplementary material for: Iron Status is Associated with Asthma and Lung Function in US Women
Source: PLoS One. 2015 Feb 17;10(2):e0117545. doi: 10.1371/journal.pone.0117545 (PMC4331366; doi:10.1371/journal.pone.0117545)
Supplement: S7 Table — Adjusted for race/ethnicity, age, smoking, income, and BMI. Anemia was defined as a hemoglobin <12.0 mg/dL and iron deficiency was defined as ferritin <20 ng/mL. §n = 2193; †n = 2274. (DOCX) [file pone.0117545.s007.docx]

**Table S7.** Relationships between iron deficiency, anemia, lung function and inflammation.

|  | | **FEV_1_/FVC ratio^§^** | **Log_10_(FeNO)^†^** |
| --- | --- | --- | --- |
|  | **Iron-status** | β (95% CI) | β (95% CI) |
| **No anemia** | Sufficient | -ref- | -ref- |
|  | Deficient | -0.006 (-0.01 to 0.001) | -0.02 (-0.06 to 0.02) |
| **Anemia** | Sufficient | -0.007 (-0.03 to 0.01) | -0.06 (-0.1 to 0.03) |
|  | Deficient | -0.0007 (-0.01 to 0.01) | -0.007 (-0.07 to 0.05) |

Adjusted for race/ethnicity, age, smoking, income, and BMI

Anemia was defined as a hemoglobin <12.0 mg/dL and iron deficiency was defined as ferritin <20 ng/mL

^§^n=2193; ^†^n=2274
