# Supplementary material for: Iron Status is Associated with Asthma and Lung Function in US Women
Source: PLoS One. 2015 Feb 17;10(2):e0117545. doi: 10.1371/journal.pone.0117545 (PMC4331366; doi:10.1371/journal.pone.0117545)
Supplement: S8 Table — *Adjusted for race/ethnicity, age, smoking, income, and BMI. Bolded results are statistically significant, with p<0.05. ‡n = 1046 for unadjusted, n = 930 for adjusted; Q1–4: 1.8–48.0ng/ml, Q5: >48.0ng/ml. §ferritin restricted to values from 20 to 300 ng/ml, inclusive; n = 693 for unadjusted, n = 621 for adjusted. †n = 1043 for unadjusted, n = 927 for adjusted. (DOCX) [file pone.0117545.s008.docx]

**Table S8.** (age 12 to 19) Relationships between iron status and asthma outcomes.

|  | **Lifetime Asthma** | | **Current Asthma** | | **Asthma Attack/Episode in Past Year** | |
| --- | --- | --- | --- | --- | --- | --- |
|  | Unadjusted | Adjusted* | Unadjusted | Adjusted* | Unadjusted | Adjusted* |
|  | OR (95% CI) | | OR (95% CI) | | OR (95% CI) | |
| **Ferritin Iron Indices (higher levels indicative of *more* iron)** | | | | | | |
| Log_10_(ferritin)^‡^ | 0.96 (0.56 to 1.64) | 0.96 (0.55 to 1.65) | 1.16 (0.56 to 2.39) | 1.20 (0.54 to 2.69) | 0.59 (0.24 to 1.49) | 0.55 (0.23 to 1.33) |
| Log_10_(ferritin)^§^_(20-300 ng/ml)_ | 1.48 (0.43 to 5.08) | 1.29 (0.33 to 5.09) | 4.33 (0.98 to 19.24) | 4.15 (0.70 to 24.62) | 1.59 (0.17 to 15.09) | 0.75 (0.08 to 7.13) |
| Ferritin, quintile 5 vs. quintiles 1-4^‡^ | 1.20 (0.76 to 1.90) | 1.12 (0.67 to 1.87) | 1.54 (0.87 to 2.71) | 1.45 (0.71 to 2.97) | 1.17 (0.50 to 2.71) | 0.89 (0.36 to 2.20) |
| **Serum Transferrin Receptor Iron Indices (higher levels indicative of *less* iron)** | | | | | | |
| Log_10_(sTFR)^†^ | 1.50 (0.55 to 4.06) | 1.34 (0.41 to 4.42) | 1.66 (0.50 to 5.51) | 1.41 (0.29 to 6.83) | 4.45 (1.04 to 19.13) | **6.13 (1.16 to 32.43)** |
| Log_10_(sTfR/log_10_ ferritin)^†^ | 1.21 (0.60 to 2.42) | 1.14 (0.53 to 2.45) | 1.14 (0.46 to 2.83) | 1.02 (0.33 to 3.09) | 2.61 (0.97 to 7.00) | **2.97 (1.01 to 8.71)** |

*Adjusted for race/ethnicity, age, smoking, income, and BMI

**Bolded** results are statistically significant, with p<0.05

^‡^n=1046 for unadjusted, n=930 for adjusted; Q1-4: 1.8-48.0ng/ml, Q5: >48.0ng/ml

^§^ferritin restricted to values from 20 to 300 ng/ml, inclusive; n=693 for unadjusted, n=621 for adjusted

^†^n=1043 for unadjusted, n=927 for adjusted
